# Supplementary material for: Prepartum Magnesium Butyrate Supplementation of Dairy Cows Improves Colostrum Yield, Calving Ease, Fertility, Early Lactation Performance and Neonatal Vitality
Source: Animals (Basel). 2023 Apr 12;13(8):1319. doi: 10.3390/ani13081319 (PMC10135157; doi:10.3390/ani13081319)
Supplement: Supplementary file 1 [file animals-13-01319-s001.zip › Supplementary File S1_THI.pdf]

Supplementary Table S1

Average temperature humidity index (THI) values and the number of animals enrolled in the study during the study period in the barns of magnesium butyrate (MgB) and Control groups.

Month	Average THI		No. of animals enrolled	
	Control	MgB	Control	MgB
February	42	40	10	11
March	47	47	21	24
April	50	50	24	25
May	58	58	24	26
June	70	71	14	12
July	72	72	15	14

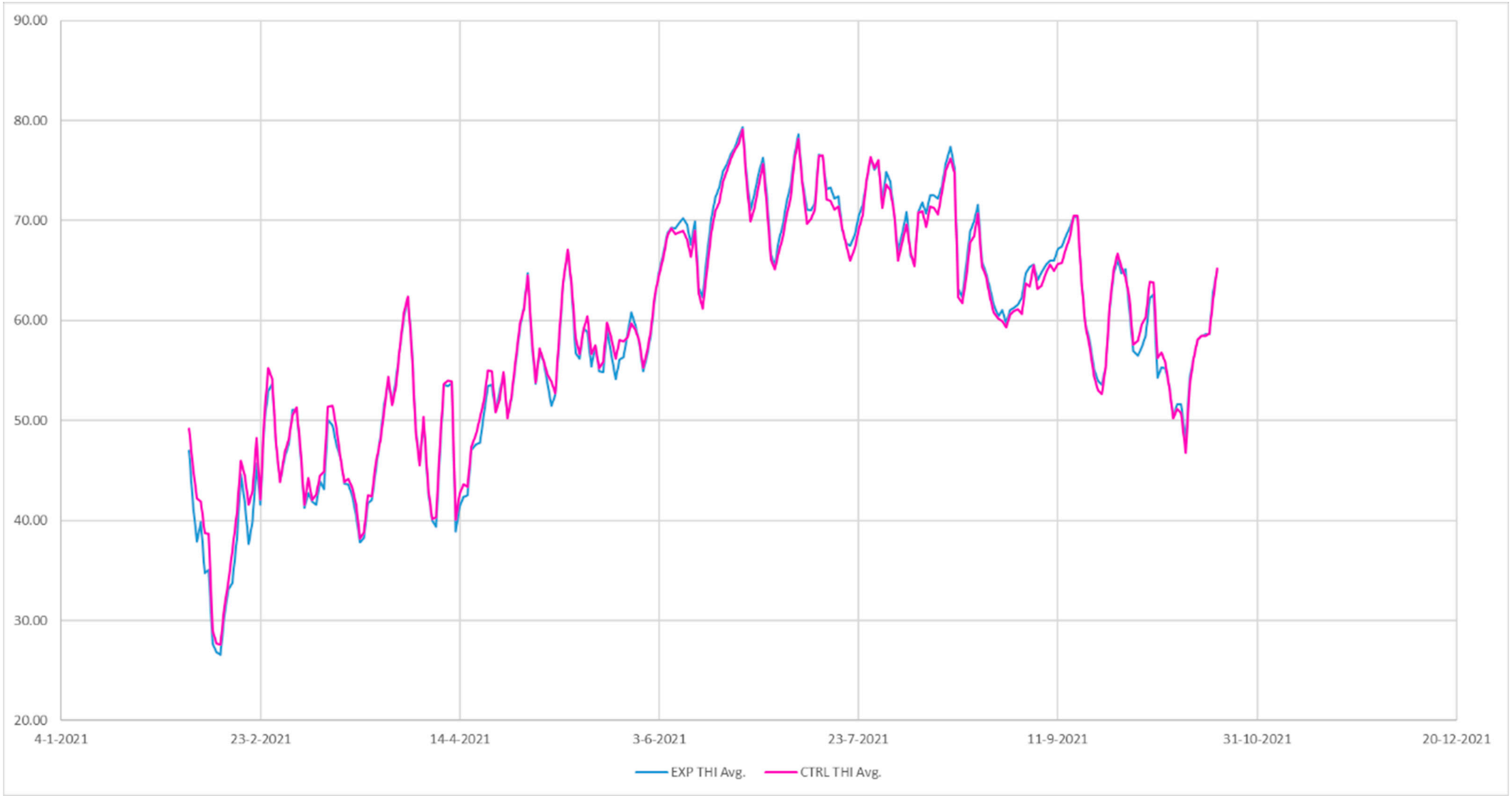

Supplementary Figure S1. Changes in average daily THI values during the study period in the barns of magnesium butyrate (EXP) and Control (CTRL) groups.

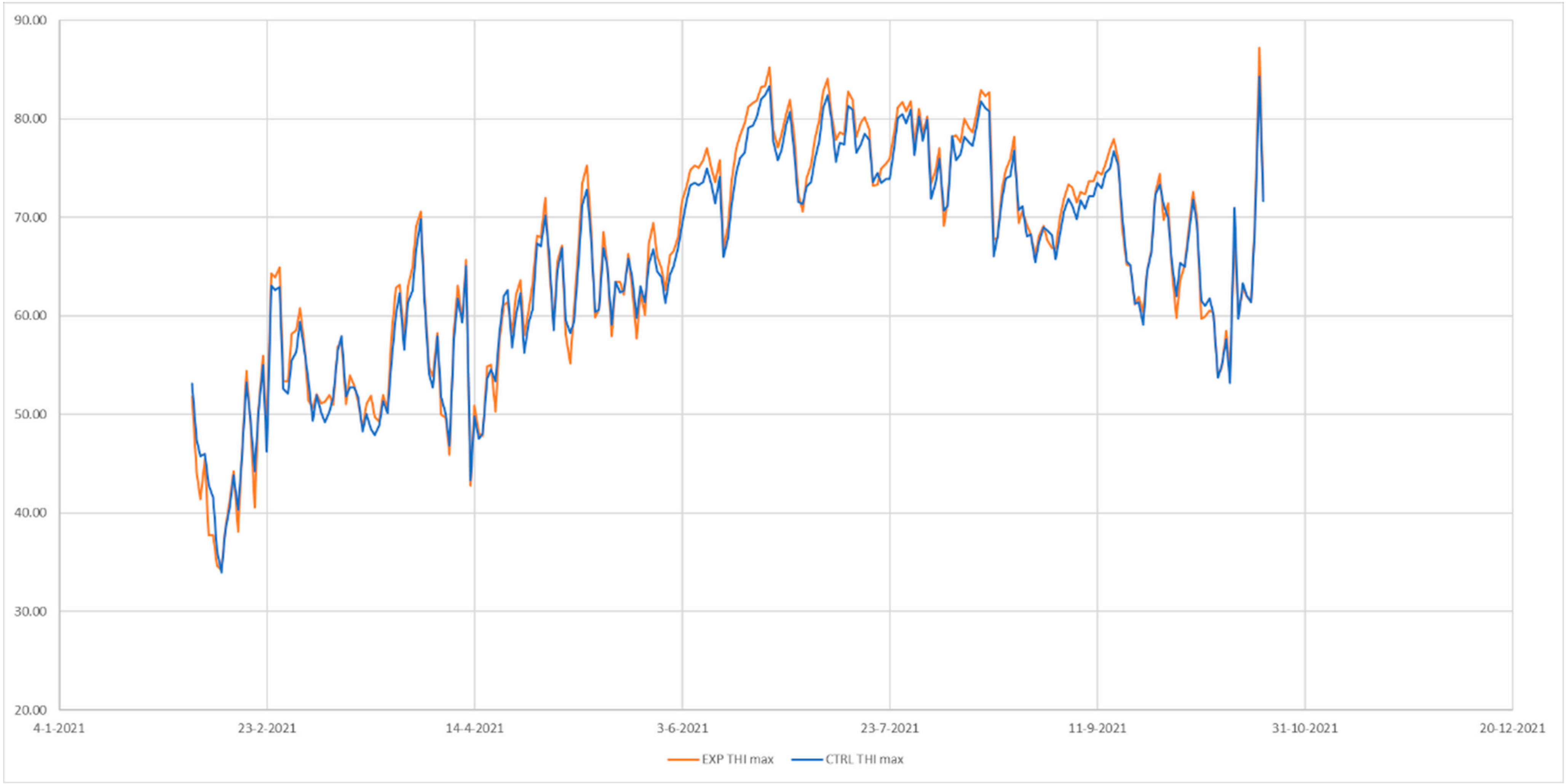

Supplementary Figure S2. Changes in maximal daily THI values during the study period in the barns of magnesium butyrate (EXP) and Control (CTRL) groups.
