# Supplementary material for: Prepartum Magnesium Butyrate Supplementation of Dairy Cows Improves Colostrum Yield, Calving Ease, Fertility, Early Lactation Performance and Neonatal Vitality
Source: Animals (Basel). 2023 Apr 12;13(8):1319. doi: 10.3390/ani13081319 (PMC10135157; doi:10.3390/ani13081319)
Supplement: Supplementary file 1 [file animals-13-01319-s001.zip › Supplementary File S2_Cowparitydistribution.pdf]

Supplementary Table S2

Number of cows enrolled into the study according to parity and experimental group (Control and Magnesium butyrate), and the percentage of cows for each parity within a group, are indicated.

Parity	No. of cows (% of group)	
	Control	Magnesium butyrate
2	45 (40.2)	41 (38.3)
3	39 (34.8)	50 (46.7)
4	12 (10.7)	11 (10.3)
5	6 (5.4)	2 (1.9)
6	4 (3.6)	3 (2.8)
7	6 (5.4)	0 (0)
