# Supplementary material for: Prepartum Magnesium Butyrate Supplementation of Dairy Cows Improves Colostrum Yield, Calving Ease, Fertility, Early Lactation Performance and Neonatal Vitality
Source: Animals (Basel). 2023 Apr 12;13(8):1319. doi: 10.3390/ani13081319 (PMC10135157; doi:10.3390/ani13081319)
Supplement: Supplementary file 1 [file animals-13-01319-s001.zip › Supplementary File S3_Lameness.pdf]

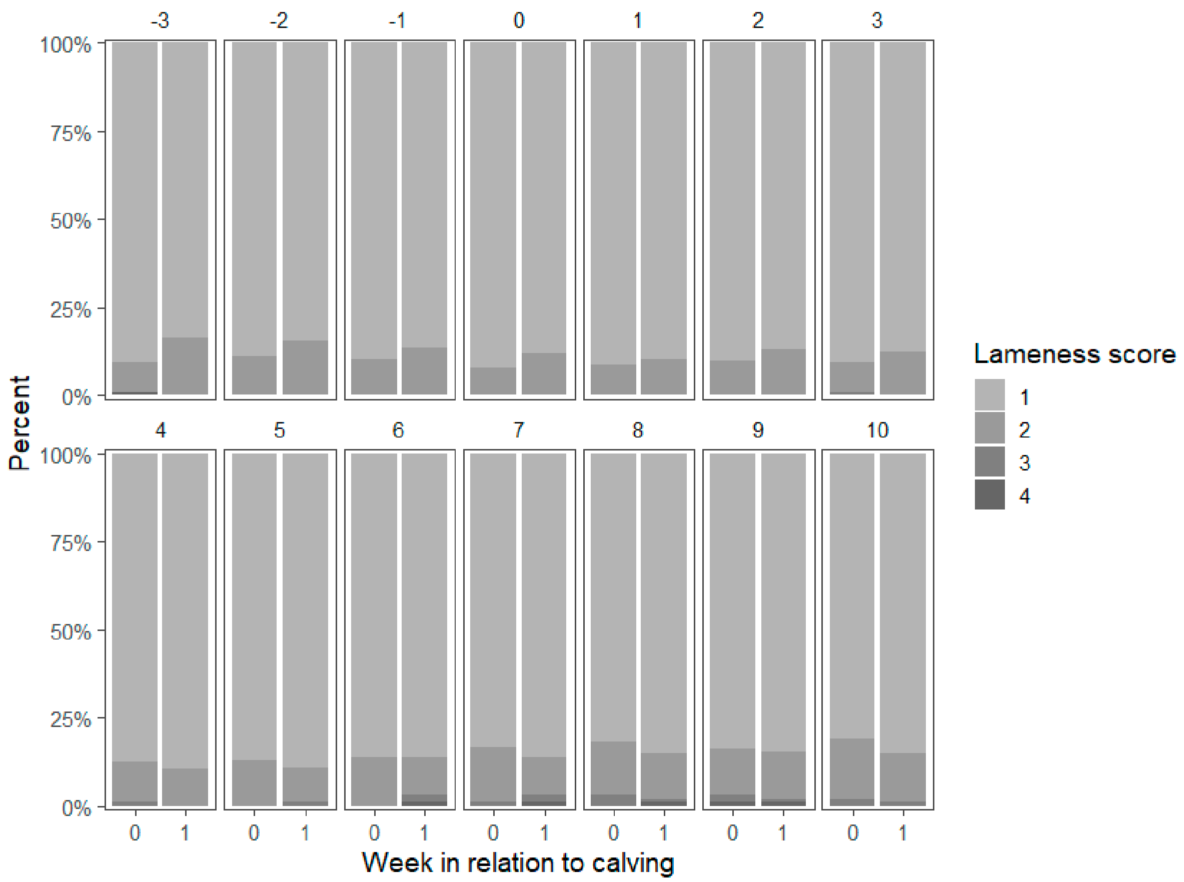

Supplementary Figure S3. Lameness score. Trial groups are indicated as: 0=Control; 1=Magnesium Butyrate.

Supplementary Table S3

Results of statistical test regarding difference in the distribution of lameness categories between magnesium butyrate and Control groups during the experiment.

Week	-3	-2	-1	0	1	2	3	4	5	6	7	8	9	10
P-value*	0.821	0.821	0.821	0.821	0.892	0.821	0.821	0.892	0.821	0.821	0.821	0.821	1	0.845

**P*-values were adjusted for multiple comparisons, by controlling for false discovery rate.
