# Supplementary material for: Prepartum Magnesium Butyrate Supplementation of Dairy Cows Improves Colostrum Yield, Calving Ease, Fertility, Early Lactation Performance and Neonatal Vitality
Source: Animals (Basel). 2023 Apr 12;13(8):1319. doi: 10.3390/ani13081319 (PMC10135157; doi:10.3390/ani13081319)
Supplement: Supplementary file 1 [file animals-13-01319-s001.zip › Supplementary File S4-milk yield_BCS.pdf]

Supplementary Table S4

Estimated average weekly milk yield in magnesium butyrate (MgB) and Control groups and standard error of mean (Linear mixed-effects model).

	Weeks											*P*-values		
	1	2	3	4	5	6	7	8	9	10	SEM	Group	Week	Group × Week
Control	27.39	34.79	35.67	38.61	38.33	37.78	38.26	34.98	36.29	36.24	0.94	0.181	<0.0001	0.0005
MgB	32.07	36.55	37.51	39.13	37.88	37.74	38.15	35.76	36.32	36.43				
P-value	<0.0001	0.057	0.048	0.581	0.636	0.962	0.911	0.418	0.975	0.841				

Supplementary Table S5

Estimated marginal means of BCS in magnesium butyrate (MgB) and Control groups and standard error of the mean (Linear mixed effects model).

	Weeks															*P*-values		
	-3	-2	-1	0	1	2	3	4	5	6	7	8	9	10	SEM	Group	Week	Group × Week
Control	3.61	3.60	3.62	3.61	3.57	3.53	3.49	3.45	3.39	3.33	3.29	3.22	3.18	3.13	0.030	0.034	<0.0001	<0.00001
MgB	3.62	3.60	3.63	3.62	3.62	3.59	3.55	3.52	3.47	3.43	3.38	3.30	3.25	3.18				
P-value	0.828	0.975	0.729	0.542	0.111	0.078	0.047	0.020	0.005	<0.001	0.003	0.011	0.041	0.101				
